# Pedicle screw versus hybrid posterior instrumentation for dystrophic neurofibromatosis scoliosis

**DOI:** 10.1097/MD.0000000000006977

**Published:** 2017-06-02

**Authors:** Jr-Yi Wang, Po-Liang Lai, Wen-Jer Chen, Chi-Chien Niu, Tsung-Ting Tsai, Lih-Huei Chen

**Affiliations:** a Department of Orthopedic Surgery, Shin Kong Wu Ho-Su Memorial Hospital, Taipei; b Department of Orthopedic Surgery, Chang Gung Memorial Hospital, Chang Gung University College of Medicine; c Bone and Joint Research Center, Chang Gung Memorial Hospital, Taoyuan, Taiwan.

**Keywords:** kyphoscoliosis, kyphosis, neurofibromatosis, scoliosis, spinal fusion

## Abstract

Surgical management of severe rigid dystrophic neurofibromatosis (NF) scoliosis is technically demanding and produces varying results. In the current study, we reviewed 9 patients who were treated with combined anterior and posterior fusion using different types of instrumentation (i.e., pedicle screw, hybrid, and all-hook constructs) at our institute.

Between September 2001 and July 2010 at our institute, 9 patients received anterior release/fusion and posterior fusion with different types of instrumentation, including a pedicle screw construct (n = 5), a hybrid construct (n = 3), and an all-hook construct (n = 1). We compared the pedicle screw group with the hybrid group to analyze differences in preoperative curve angle, immediate postoperative curve reduction, and latest follow-up curve angle.

The mean follow-up period was 9.5 ± 2.9 years. The average age at surgery was 10.3 ± 3.9 years. The average preoperative scoliosis curve was 61.3 ± 13.8°, and the average preoperative kyphosis curve was 39.8 ± 19.7°. The average postoperative scoliosis and kyphosis curves were 29.7 ± 10.7° and 21.0 ± 13.5°, respectively. The most recent follow-up scoliosis and kyphosis curves were 43.4 ± 17.3° and 29.4 ± 18.9°, respectively. There was no significant difference in the correction angle (either coronal or sagittal), and there was no significant difference in the loss of sagittal correction between the pedicle screw construct group and the hybrid construct group. However, the patients who received pedicle screw constructs had significantly less loss of coronal correction (*P* < .05). Two patients with posterior instrumentation, one with an all-hook construct and the other with a hybrid construct, required surgical revision because of progression of deformity.

It is difficult to intraoperatively correct dystrophic deformity and to maintain this correction after surgery. Combined anterior release/fusion and posterior fusion using either a pedicle screw construct or a hybrid construct provide similar curve corrections both sagittally and coronally. After long-term follow-up, sagittal correction was maintained with both constructs. However, patients treated with posterior instrumentation using pedicle screw constructs had significantly less loss of coronal correction.

## 1 Introduction

Neurofibromatosis (NF) is the most common single-gene disorder,^[1]^ affecting approximately 1 in 3000 people. Although it is an autosomal dominant hereditary disease, about half of the cases can be attributed to spontaneous mutation.^[2–4]^ In NF, cells derived from neural crest cells are affected, resulting in a variety of characteristic lesions, such as café-au-lait macules, neurofibromas, and Lisch nodules, as well as various skeletal abnormalities, of which thoracic scoliosis is the most prevalent.^[5–7]^ NF can be divided into two subtypes, peripheral neurofibromatosis (NF-1) and central neurofibromatosis (NF-2), based on clinical manifestations.^[6]^ NF-2 has been associated with bilateral acoustic Schwannomas as well as rare orthopedic manifestations.^[7]^

Spinal deformity affects 10% to 60% of NF-1 patients^[8,9]^ and is notorious for being severe and very difficult to treat. Such deformities are generally classified as either non-dystrophic or dystrophic curves based on the absence or presence of skeletal dysplasia. Non-dystrophic curves have a more benign course that is similar to idiopathic scoliosis.^[1,9]^ Dystrophic curves can be characterized by vertebral scalloping, rib penciling, spindling of the transverse processes, wedging of one or more vertebral bodies, paraspinal or intraspinal soft tissue masses, a short curve with significant apical rotation, occasionally leading to subluxed or dislocated vertebral bodies, foraminal enlargement, and defective pedicles. Early surgical intervention with anterior and posterior spinal fusion and meticulous bone grafting produces the most reliable results.^[7,10,11]^ However, a loss of reduction after anterior and posterior fusion is not rare.

In the current study, we presented our experiences in using combined anterior fusion and posterior fusion for dystrophic NF-1 scoliosis with different types of instrumentation (i.e., pedicle screw, hybrid, or all-hook constructs). We hypothesized that patients treated with pedicle screw constructs would have better correction angles and less loss of correction in both the sagittal and coronal curves.

## 2 Patients and methods

The current study used a retrospective design and analyzed a consecutive series of patients with dystrophic NF-1 who received anterior fusion and posterior instrumented fusion at our institute. This study was approved by the Institute Review Board (CGMH 201600104B0) of our hospital. The records and radiographs of all patients diagnosed with NF-1 and associated scoliosis between Sep 2001 and Jul 2010 at our institute were reviewed. The following inclusion criteria were used: (1) patients who fulfilled the criteria of NF^[12]^ with typical dystrophic scoliosis who had undergone surgical treatment, (2) patients who received combined anterior and posterior fusion with instrumentation, (3) patients who achieved bone maturity before the last follow-up. Bone maturity was defined as Risser grade 5^[13]^ at the latest follow-up.

Clinical examination included a thorough neurologic examination and assessment of curve flexibility. Radiologic examination included whole spine plain x-rays; coronal and sagittal curves were measured using Cobb technique. The Cobb angles were measured by a semi-automated digitized computer system, picture archiving and communication system (PACS), which calculating the angles by a line drawn along the upper and lower endplate of the upper-end and lower-end vertebrae.

All patients received combined anterior release/fusion followed by posterior correction/instrumentation. All the operations were performed by the senior author under somatosensory evoked potentials monitoring. The procedure was performed in 2 sessions for 4 patients and as a single-staged procedure for 5 patients. The anterior release included as many levels as possible, with an average of 4.5 levels (range, 4–5 levels) centered over the apex of the deformity. The apical vertebrae were approached from the convex side using a standard approach. Anterior fusion was performed by removing the intervertebral discs; intervertebral disc space was filled with autogenous rib grafts.

**Figure 1:**
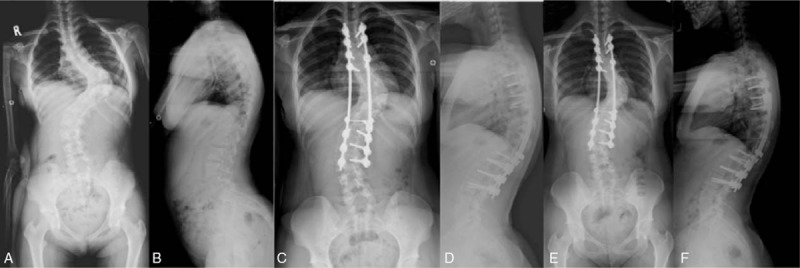
Pedicle screw construct. The pre-op (A and B), immediate post-op (C and D), and the 9-year follow-up (E and F) images.

**Figure 2:**
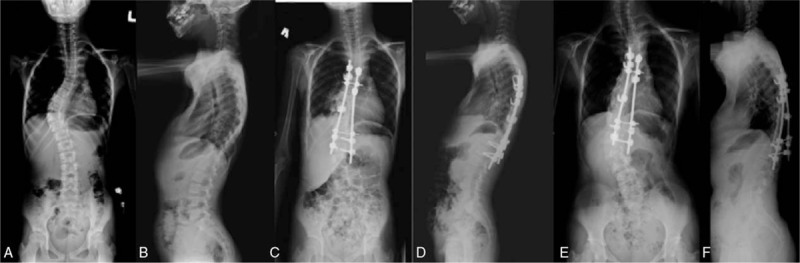
Hybrid construct. The pre-op (A and B), immediate post-op (C and D), and the 5-year follow-up (E and F) images, before revision to pedicle screw construct.

**Figure 3:**
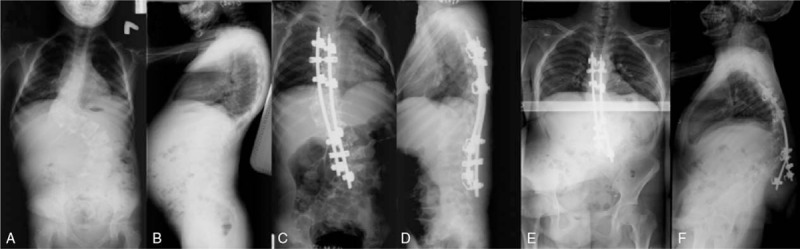
All-hook construct. The pre-op (A and B), immediate post-op (C and D), and the 12-year follow-up (E and F) images, before revision to pedicle screw construct.

Posteriorly, extreme care was taken during exposure due to occasional thinning of the laminae. Meticulous decortication was performed as well as generous autografting using iliac crest autografts. Gradual correction was performed using a combination of translation/derotation maneuvers. Posterior spinal fusion instrumentation included pedicle screw constructs (pedicle screws at both ends with/without a supplementary hook around the curve apex area), hybrid constructs (thoracic hooks and lumbar pedicle screws), and all-hook constructs (Figs. 1–3).

Postoperatively, all patients were braced for 3 months. Preoperative, postoperative, and latest follow-up coronal plane deformity and sagittal plane deformity were collected. Loss of curve correction was defined as the angle of the latest curve minus the angle of the immediate postoperative curve.

We used the Mann–Whitney *U* test to evaluate differences in preoperative curve angle, immediate postoperative curve reduction, and latest follow-up loss of correction between the pedicle screw group and the hybrid group. Statistical analysis was performed using SPSS 20.0 software (SPSS Inc, Chicago, IL). Probability (*P*) values less than 0.05 were considered statistically significant.

## 3 Results

Between September 2001 and July 2010, 12 patients were diagnosed with dystrophic NF at our institute. Three of these patients were not included in our study: 2 received growing rod systems, and the other received posterior instrumentation only. Therefore, in total, 9 patients were enrolled in the study (Table 1).

**Table 1:**
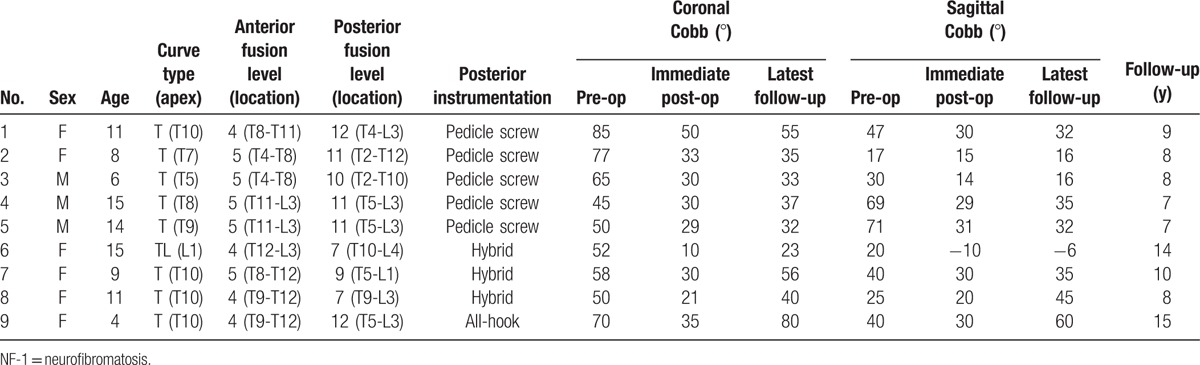
Summary of clinical data obtained in 9 patients with NF-1 dystrophic scoliosis.

The following types of posterior instrumentation were used: pedicle screw constructs (n = 5), hybrid constructs (n = 3), and an all-hook construct (n = 1). Postoperatively, all the patients had intact neurologic status. There was no intraoperative neurovascular injury, incidental durotomy, or postoperative infection.

The study cohort included 6 female patients and 3 male patients. The mean follow-up period was 9.5 ± 2.9 years. The average age at surgery was 10.3 ± 3.9 years. Eight patients had thoracic-type curves, and one patient had a thoracolumbar type curve. All of the patients had typical dystrophic curves and at least 3 of the 5 following criteria: vertebral scalloping, penciling of the ribs, severe apical vertebral rotation, spindled transverse processes, and foraminal enlargement.^[14]^

Preoperatively, the average scoliosis curve was 61.3 ± 13.8°, and the average kyphosis curve was 39.8 ± 19.7°. Postoperatively, the scoliosis and kyphosis curves were reduced to 29.7 ± 10.7° and 21.0 ± 13.5°, respectively. The latest follow-up scoliosis and kyphosis curves were 43.4 ± 17.4° and 29.4 ± 18.9°, respectively.

Among the patients treated with the pedicle screw construct, the mean coronal and sagittal Cobb angles were 64.4 ± 17.0° and 46.8 ± 23.7° before surgery, 34.4 ± 8.8° and 23.8 ± 8.5° after surgery, and 38.4 ± 9.5° and 26.2 ± 9.4° at the latest follow-up, respectively. The postoperative coronal curve and sagittal curve corrections were 30 ± 11.7° and 23.0 ± 16.6°, respectively. The mean correction loss was 4.0 ± 2.0° in the coronal plane and 2.4 ± 2.1° in the sagittal plane.

For the patients treated with the hybrid construct, the mean coronal and sagittal Cobb angles were 53.3 ± 4.2° and 28.3 ± 10.4° before surgery, 20.3 ± 10.0° and 13.3 ± 20.8° after surgery, and 39.7 ± 16.5° and 24.6 ± 27.0° at the latest follow-up, respectively. The postoperative coronal curve and sagittal curve corrections were 33.0 ± 7.8° and 13.2 ± 15°, respectively. The mean correction loss was 19.3 ± 6.5° in the coronal plane and 11.3 ± 11.8° in the sagittal plane. One patient (No. 7) required revision surgery due to progression of deformity 5 years after initial surgery. The second surgery for the patient used posterior instrumentation with a pedicle screw construct.

One patient (No. 9) was treated using an all-hook construct. For this patient, the coronal and sagittal Cobb angles were 70° and 40° before surgery, 35° and 30° after surgery, and 80° and 60° at the latest follow-up, respectively. The postoperative coronal curve and sagittal curve corrections were 35° and 10°, respectively. The correction loss was 45° in the coronal plane and 30° in the sagittal plane. The distal lumbar hooks were dislodged. The patient required revision surgery due to progressive trunk deformity 12 years after initial surgery. The patient received posterior instrumentation with a hybrid construct.

**Figure 4:**
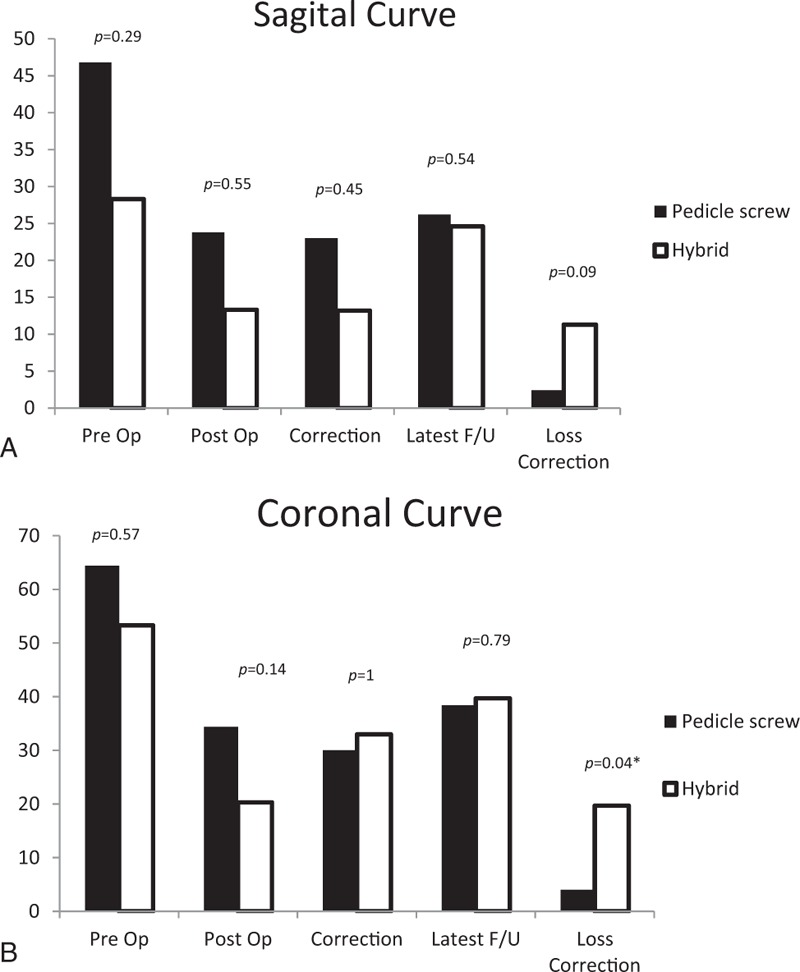
(A) No significant differences were found in preoperative or postoperative correction angle and latest follow-up curve angle between the pedicle screw construct group and the hybrid construct group in sagittal alignment. (B) A significant difference was found with regard to loss of coronal correction between the 2 groups.

There were no significant differences between the pedicle screw construct group and the hybrid construct group in either coronal or sagittal Cobb angles preoperatively (*P* = .66 and *P* = .3) or postoperatively (*P* = .17 and *P* = .55). Additionally, there were no significant differences in coronal and sagittal curve correction angles between these 2 groups (*P* = .88 and *P* = .45). However, the patients who were treated with the pedicle screw construct had significantly less loss of coronal correction than the hybrid construct group (*P* < .05). In contrast, there was no significant difference in the loss of sagittal curve correction between these 2 groups (Fig. 4).

## 4 Discussion

Spinal deformity caused by NF is very difficult to treat. In typical scoliosis clinics, only 3% of patients have NF, and the percentage of those with dystrophic type deformities is even less.^[11]^ Thus, the personal experience of any single spinal surgeon with such a presentation would not be extensive.^[15]^ Dystrophic curves cannot be corrected with bracing.^[7,16]^ Thus, the need for early intervention is well established. Circumferential fusion via dual-incision anterior and posterior approaches yields the most reliable results and has been recommended by many authors.^[10,16]^ However, there is a high incidence of pseudoarthrosis (20–33%) and strong tendency for further progression of the deformity even after anterior and posterior fusion.^[17–19]^ This is due to the progressive erosion of the fusion mass after surgery.^[17–19]^ As such, surgical re-exploration and repeated bone grafting after 6 months has been advocated for the treatment of pseudoarthrosis.^[1,20]^

The first implant used for thoracic spine surgical treatment of scoliosis was Harrington rod, detailed in a report published in 1962.^[21]^ This device comprised a non-segmental hook and rod system and produced disappointing results: several authors reported poor outcomes when using Harrington rod for the treatment of dystrophic NF scoliosis. The incidence of pseudoarthrosis was 38% to 64% when treated with posterior instrumented fusions only.^[7,16,22]^ If additional anterior fusion was performed, a fusion rate of 77% to 80% was noted.^[7,16]^

The multiple segmental hook and rod system developed by Cotrel and Dubousset served as a milestone for scoliosis surgery.^[23]^ In a report by Holt and Johnson,^[24]^ 5 NF scoliosis patients were treated with Cotrel-Dubousset instrumentation (CDI): 3 cases (60%) exhibited progression of scoliosis despite the use of CDI and fusions, and 1 case (20%) required revision surgery. In another report, Shufflebarger^[25]^ reviewed the records of 12 patients treated with CDI and noted excellent results with minimal loss of correction and no pseudoarthrosis. However, the majority of these cases were non-dystrophic. In our series, only 1 case received anterior fusion and posterior fixation with a multiple segmental hook and rod system (No. 9). Progressive deformity was noted during follow-up, and revision surgery with posterior fusion using a hybrid construct was performed 12 years after the initial fusion surgery.

Pedicle screw fixation is regarded as a significant improvement over conventional spinal stabilization constructs using wires, hooks, or both. It offers better vertebral grip with 3-column purchase and an advantageous moment arm because of the anatomic placement of the pedicle screws compared with hook placement on the lamina.^[26]^ Pedicle screws are commonly used in the lumbar spine and are familiar to most spinal surgeons. However, it is generally agreed that the placement of thoracic pedicle screws is technically demanding, and the risk of spinal cord and nerve injury significantly increases if the screws are placed by surgeons lacking experience and proper training. Hybrid constructs with thoracic hooks and lumbar pedicle screws with and without sublaminar wire have also been used for the treatment of NF scoliosis. These constructs take advantage of the stronger holding power provided by pedicle screws while avoiding the placement of pedicle screws directly into deformed thoracic curves. Koptan and ElMiligui^[14]^ reported the use of extensive and vigorous anterior release with posterior hybrid instrumentation for managing server dystrophic neurofibromatosis. They advocated that sublaminar wires allow safe gradual correction and even distribution of forces over multiple anchor points improving the correction achieved and decreasing implant-related complications. However, the potential risk of sublaminar wire cutting through the thin lamiae of NF spine remained an issue. Li et al^[27]^ reported the results from 19 cases who were over 10 years in age and whose scoliosis was <90°. These patients received posterior fusion with rostral hooks and distal pedicle screws without an anterior procedure. Following surgery, there were no cases of coronal or sagittal decompensation, neurologic complications, or infections. There were 8 (42.1%) complications, and pseudoarthrosis with instrumentation failure that required revision surgery occurred in 1 patient (5.2%).^[27]^ Among the 3 cases receiving posterior fusion with hybrid constructs in our study, 1 case required revision surgery using a pedicle screw construct due to progression of spinal deformity.

The safety and efficacy of thoracic pedicle screw placement has been established since early 2000. Suk et al^[28]^ reported results from 462 patients with spinal deformities who received thoracic pedicle screw fixation. Screw-related neurologic complications occurred in 4 patients (0.8%). The deformity correction was 69.9% for idiopathic scoliosis and 60.7% for congenital scoliosis. The sagittal plane deformity correction was 47° for kyphosis. The authors concluded that thoracic pedicle screw fixation is a reliable method of treating spinal deformities, with excellent deformity correction and a high margin of safety. The treatment of idiopathic scoliosis using a pedicle screw technique is a well-established procedure. However, only a few case series have presented clinical results regarding the treatment of dystrophic NF scoliosis using pedicle screw constructs.^[29–31]^ The dysplasia of pedicles increased the challenge of pedicle screw insertion.

In the current study, we reported the results of 9 cases of dystrophic NF-1 scoliosis surgically treated with anterior release/fusion and posterior fusion with instrumentation at our institute. Similar anterior fusion techniques and fusion levels were used with different types of posterior instrumentation (e.g., a pedicle screw construct, a hybrid construct, or an all-hook construct). Statistically, the reduction rate between the pedicle screw construct and the hybrid construct did not significantly differ in either the coronal or sagittal plane. However, the pedicle screw construct group had significantly less loss of coronal correction compared with the hybrid construct group; however, there was no significant difference in loss of sagittal correction between these 2 constructs.

Several previously published studies comparing hook, hybrid, and all-screw constructs with respect to correction rates of spinal deformities have discussed in the scenario of treating adolescent idiopathic scoliosis^[32–35]^ with posterior-only reduction and instrumentation. Lowenstein et al^[35]^ compared patients with adolescent idiopathic scoliosis who underwent isolated posterior spinal fusion and instrumentation placement. In the referenced study, 17 patients underwent fusions using all-screw constructs, and 17 patients underwent fusions using hybrid constructs. There were no significant differences between the all-screw construct group and the hybrid construct group in coronal or sagittal correction, although there was a trend toward better correction of the main thoracic curve in the all-screw construct group (*P* < .089). Di Silvestre et al^[34]^ suggested that pedicle-screw only constructs allowed for greater coronal correction of both main thoracic and secondary lumbar curves and less loss of postoperative correction, and they required fewer revision surgeries. However, according to a meta-analysis conducted by Cao et al^[36]^ in 2014, there is an overall tendency for both types of instrumentation to restore thoracic kyphosis. Hybrid constructs seem to be more powerful in restoring kyphosis than pedicle screw constructs. Our current series compared surgical outcomes in patients who received standard anterior release/fusion followed by posterior instrumentation with these 2 types of construct and found no significance differences in correction rates. The anterior fusion procedure used in this study decreased the role of posterior correction, and the placement of posterior instrumentation primarily maintained the reduction following anterior fusion. However, after an average of 9.5 years of follow-up, a significant loss of coronal correction was noted in the hybrid construct group. This relationship may due to the well-known crankshaft phenomenon, in which a loss of coronal deformity correction occurs in patients secondary to continued anterior column growth.^[37]^ The risk of developing curve progression after fusion secondary to anterior growth has been shown to decrease following the placement of pedicle screw instrumentation.^[38,39]^

Recently, evidence supporting the use of posterior vertebral column resection (pVCR) with pedicle screw constructs for severe deformity has been reported.^[29,40,41]^ Although excellent correction was achieved, the potential risk for spinal cord injury following pVCR due to distraction, compression, and translation as a result of deformity correction, kinking, or dural buckling in response to spinal shortening and direct manipulation of the spinal cord remains a major concern.^[42–44]^ Higher rates of general and neurologic complications have been reported following the use of a posterior-only approach versus a combined anterior and posterior approach.^[45]^ Thus, posterior-only techniques must be reserved for only the most severe and rigid of deformities and should only be executed by a highly experienced surgical team.

The main limitations of this study include its retrospective nature and limited patient numbers. Further prospective, random, comparative studies can provide stronger scientific evidence, and multiple center studies to increase patient numbers for more thorough statistical analysis are still needed.

## 5 Conclusions

It is difficult to intraoperatively correct dystrophic deformities and to maintain such correction after surgery. Combined anterior release/fusion and posterior fusion using either a pedicle screw construct or a hybrid construct provide similar curve correction both sagittally and coronally. After long-term follow-up (average, 9.5 years), sagittal correction was still maintained in both construct types. However, the patients who were treated with posterior fusion using pedicle screw constructs had significantly less loss of coronal correction compared with those treated with the hybrid and all-hook constructs. We suggest using pedicle screw constructs to maintain correction following anterior release/fusion of NF dystrophic kyphoscoliosis.
